# Identification of approximate symmetries in biological development

**DOI:** 10.1098/rsta.2020.0273

**Published:** 2021-12-27

**Authors:** Punit Gandhi, Maria-Veronica Ciocanel, Karl Niklas, Adriana T. Dawes

**Affiliations:** ^1^ Department of Mathematics and Applied Mathematics, Virginia Commonwealth University, Richmond, VA, USA; ^2^ Department of Mathematics and Department of Biology, Duke University, Durham, NC, USA; ^3^ School of Integrative Plant Biology, Cornell University, Ithaca, NY, USA; ^4^ Department of Mathematics and Department of Molecular Genetics, The Ohio State University, Columbus, OH, USA

**Keywords:** approximate symmetries, asymmetry measure, information theory and entropy, morphogenesis

## Abstract

Virtually all forms of life, from single-cell eukaryotes to complex, highly differentiated multicellular organisms, exhibit a property referred to as symmetry. However, precise measures of symmetry are often difficult to formulate and apply in a meaningful way to biological systems, where symmetries and asymmetries can be dynamic and transient, or be visually apparent but not reliably quantifiable using standard measures from mathematics and physics. Here, we present and illustrate a novel measure that draws on concepts from information theory to quantify the degree of symmetry, enabling the identification of approximate symmetries that may be present in a pattern or a biological image. We apply the measure to rotation, reflection and translation symmetries in patterns produced by a Turing model, as well as natural objects (algae, flowers and leaves). This method of symmetry quantification is unbiased and rigorous, and requires minimal manual processing compared to alternative measures. The proposed method is therefore a useful tool for comparison and identification of symmetries in biological systems, with potential future applications to symmetries that arise during development, as observed *in vivo* or as produced by mathematical models.

This article is part of the theme issue ‘Recent progress and open frontiers in Turing’s theory of morphogenesis’.

## 1. Introduction: symmetry in biological and mathematical contexts

Humans seem to have an inherent ability to identify and appreciate symmetry throughout the natural world, even in the absence of a formal definition. We see symmetry in virtually all forms of life, from single-cell eukaryotes like *Chlamydamonas* [1] to complex, highly differentiated multicellular organisms such as ourselves [2]. Symmetry is thought to play an important role in a range of natural processes, including morphogenesis during ontogeny and growth [3], the dynamics of populations [4,5] and the functioning of ecosystems [6–8]. Symmetry has a rich history in mathematics, and underpins many fundamental theories in physics [9,10]. Group theory has become the standard language of symmetry in mathematics, where symmetries are defined in terms of transformations that leave an object of interest invariant. This precise mathematical definition of symmetry and associated measures of symmetry breaking have provided a framework for modelling the emergence of order, such as crystalline structure, in complex systems [11,12].

A general mathematical theory for the role of symmetry in the formation of spatio-temporal patterns through nonlinear dynamical processes was developed, in part, through interactions between mathematicians and experimental fluid dynamicists [13]. The specific group structure associated with the set of spatio-temporal symmetries of a given model, and very little other information, can be used to make predictions about possible symmetries exhibited by equilibrium states generated through ‘pattern forming’ instabilities [14,15]. Patterns are thus often characterized mathematically in terms of spontaneous symmetry breaking, i.e. the symmetries of the model that are *not* possessed by the patterned state.

Symmetries in models consisting of coupled differential equations typically have one or more of the following consequences, as discussed in [16]: multiplicity, characterized by multiple equilibria; symmetrically related states, which are preserved under a change of coordinates; and rhythmic patterns of synchrony in time-periodic oscillations. These symmetry characteristics can be identified in models of biological phenomena ranging from gene regulation to animal locomotion [16,17]. The formation of spatial patterns is often modelled by reaction–diffusion systems of the form proposed by Turing [18]. One such Turing model has been explored on both a periodic domain and a circular disc [19,20]. For circular domains, symmetries of steady-states are characterized in terms of the observed number of spots in the pattern [20,21]. These symmetric states are compared with early developmental patterns, such as the pentagonal symmetry associated with sea urchin morphogenesis [22]. These investigations were extended to growing surfaces, demonstrating that curvature and growth rate dictate the final spatial pattern [23]. Proposed measures for patterns have been able to successfully distinguish transitions between pattern types when control parameters are varied [24], and even identify constitutive relations that govern the dynamics of the underlying system [25].

Symmetries are often approximate in biological settings and the quantification of asymmetry can, in conjunction with mathematical models, potentially provide useful insights into underlying regulatory mechanisms. Many biological studies do not explicitly provide a definition of symmetry, but instead assume that readers understand what is meant by, for instance, bilateral symmetry in flowers [26]. Indeed, plants provide excellent model systems by manifesting unusual and robust examples of symmetry and symmetry breaking, including radial, bilateral, translation and scaling symmetries [27]. Several measures to quantify bilateral symmetry in natural objects such as flowers have been proposed. One such measure uses defined object landmarks and then quantifies variation away from these landmarks when the image is reflected across the bilateral symmetry axis [28]. However, this measure does not take into account information from non-landmark points, and the landmark points and symmetry axis must be defined manually. More recently, a symmetry measure called the Simple Indicator was proposed that quantifies the normalized area difference between object segments separated by the axis of symmetry [29,30]. Although this measure uses information about the overall shape of the object (in this case a leaf), the application has been limited to measurements of bilateral symmetry. General measures that can deal with more complex symmetries which are common in biological systems are currently not widely available.

Here, we propose a measure of asymmetry that is grounded in concepts of entropy and information theory [31], and apply it to the identification of approximate symmetries in a way that reduces user bias. Our approach is to apply the proposed Transformation Information (TI) measure to quantify asymmetries of objects under transformations parametrized by one or more continuous variables (e.g. rotation angle or location and angle of reflection axis). Local *minima* in the resulting TI function are then identified as approximate symmetries of the object. Moreover, the relative values of the TI measures at these minima provide a way to quantify deviation from perfect symmetry. While this approach can be automated, optimization of the images to enhance contrast and crop out neighbouring objects can improve identification of the approximate symmetries. We apply this measure to a number of test cases, and show that it correctly identifies fivefold symmetry in patterns produced by a Turing model, and approximate rotation and translation symmetries in organic objects including flowers and leaves. By quantifying the asymmetry of approximate symmetries in a system, this measure allows for comparison across diverse samples, and a method for identifying and quantifying changes in symmetries due to perturbation in an unbiased way.

## 2. An information theoretic measure for asymmetry

Methods for the quantification of asymmetry have appeared in a variety of contexts, ranging from automatic facial recognition [32] to quantum physics [33]. When the symmetry is perfect, the object of interest remains invariant under the associated transformation. Asymmetry measures commonly rely on some way of quantitatively comparing the object to its transformation. Klingenberg [34] provides a detailed review in the context of biological systems, whereas the review by Savriama [35] focuses specifically on floral asymmetry. One approach, originally proposed by Zabrodsky *et al.* [36,37] to quantify asymmetry in molecular structure, relies on measuring the (Euclidean) distance between certain ‘landmark points’ of the original object to those of the transformed object. Other approaches more generally seek to construct a normed vector space that provides a direction as well as magnitude of asymmetry, along with a way to measure differences in the asymmetry vector [38,39]. A difficulty with many of these approaches is that they require identification of landmarks or other features of the object of interest. Some approaches are restricted to particular types of symmetry such as bilateral symmetry [29] or rotational symmetry [38]. The method described here has the potential to be applied to images without any such identifications or restrictions.

Transformation Information has been proposed as a quantitative measure of symmetry breaking in the context of condensed matter systems [31]. It links ideas about information and entropy to symmetry, and is shown to be a generalization of other known information measures. Here we propose it as a tool for quantifying asymmetry in biological systems. The core idea behind this approach is to measure asymmetry by comparing the object of interest with its transformation under the potential symmetries of interest. Perfectly symmetric objects are unchanged under the associated transformation, and have a transformation information value of zero with respect to that symmetry.

In order to define the transformation information-based measure of asymmetry, we first construct a positive-valued function, $\mu :D\to R^{+}$, that assigns an intensity to each point on the object of interest (represented by the domain D). The magnitude of the difference between two points on the object are coded by the difference in the values of the function μ. For a two-dimensional domain D, the TI associated with a symmetry is then defined as

2.1 $$\text{TI}=\frac{1}{|\tilde{D}|}\int_{\tilde{D}}\mu (x)\ln\left[\frac{\mu (x)}{T_{a}\mu (x)}\right]\text{d}A,$$

where $T_{a}$ is the transformation associated with the symmetry of interest, $\tilde{D}$ is the intersection of the domains of the original and transformed image, and $|\tilde{D}|=\int_{\tilde{D}}\text{d}A$. Equation (2.1) has roots in the Kullback–Liebler divergence, or relative entropy, between two probability distributions p and q, given by $D_{\text{KL}}(p,q)=\sum_{i}p_{i}\ln(p_{i}/q_{i})$ [40,41]. One can interpret $D_{\text{KL}}(p,q)$ as a measure of the amount of information lost by approximating p using q. The relative entropy is often used to define a ‘distance’ between two distributions, although it is not strictly a metric since it does not satisfy the triangle inequality, $D_{\text{KL}}(p,q)\leq D_{\text{KL}}(p,r)+D_{\text{KL}}(r,q)$, and is not in general symmetric, $D_{\text{KL}}(p,q)\neq D_{\text{KL}}(q,p)$. However, when the transformation $T_{a}$ maps D onto itself, so that $D=\tilde{D}$, equation (2.1) is in fact symmetric in μ and $T_{a}\mu$.

In practice, since we apply this measure to images, we use pixel intensities as a choice for the function μ, making sure that only positive numbers are included in our pixel intensity scale. The transformations of interest do not typically map an image entirely onto itself. Some sections of the transformed image land at locations outside of the rectangular region of the original image and some sections of the original image do not overlap with the rectangular region of the transformed image. We handle this by only computing the integral in equation (2.1) over the intersection of the image and its transformation, which we denote by $\tilde{D}$. In doing so, we lose the symmetry between μ and $T_{a}\mu$ in TI, since the domain $\tilde{D}$ of integration generally contains different parts of the image and its transformation rather than all of both. For the biological images considered in this study, this means that the domain of integration may exclude parts of the image background, which we do not need to consider in assessing symmetries. Other ‘boundary conditions’ may be more suitable for dealing with the finite size of the image in certain applications, e.g. we periodically extend the domain for considering TI with respect to translation to match the periodic boundary conditions of our simulations in §5.

### (a) Sample application to Turing patterns on a disk

In appendix A, we provide an algorithm for the application of TI to identify approximate symmetry in biological images. We summarize these steps in table 1 to allow for reference in this section. Here, we start by illustrating the application of this measure to a Turing pattern on a circular domain with pentagonal symmetry. Studies [20,22] propose that such a pattern may seed more complex patterns in a multi-stage model for morphogenesis.

**Table 1.** Summary of steps from the transformation information algorithm for identifying symmetries described in appendix A.

Step (1)	process image and extract intensities μ
Step (1.1)	determine the image centre coordinates using TI
Step (2)	measure TI for rotation symmetries
Step (3)	measure TI for reflection symmetries
Step (4)	visualize TI as a function of rotation/reflection angle
Step (5)	identify approximate symmetries using TI peaks.

We consider a reaction–diffusion system, numerically studied in [20–23], that models the temporal evolution of the concentrations of two proteins or chemicals u and v with different diffusion coefficients according to the equations:

2.2 $$\frac{\partial u}{\partial t}=D_{u}\delta \nabla^{2}u+\alpha u(1-r_{1}v^{2})+v(1-r_{2}u)$$

and

2.3 $$\frac{\partial v}{\partial t}=\delta \nabla^{2}v+\beta v\left(1+\frac{\alpha r_{1}}{\beta }uv\right)+u(\gamma +r_{2}v),$$

where δ describes the size of the system and all values of the interaction parameters are provided in [22].

We use the numerical scheme proposed in [22] to solve equations (2.2) and (2.3) in polar coordinates on a two-dimensional disc domain with zero-flux boundary conditions.

**Figure 1.**
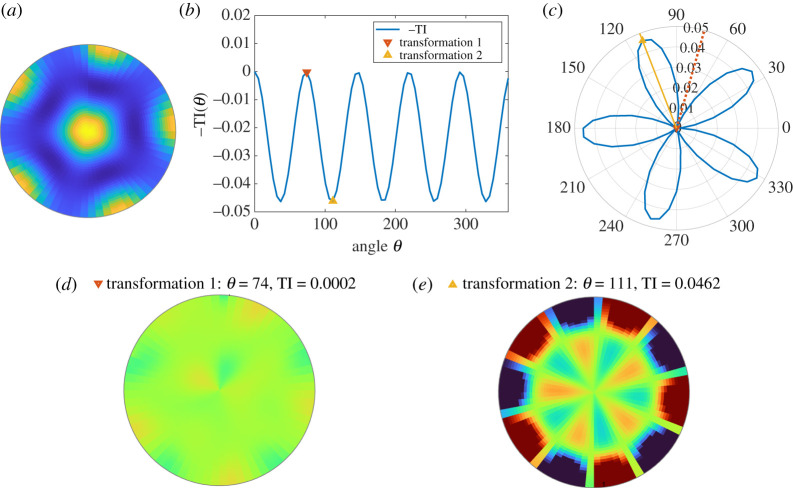
Transformation information (TI) as a measure for analysing rotation symmetries of the pentagonal Turing pattern in [20]. (*a*) Turing pentagonal pattern on a disk. (*b*) TI measure as a function of the angle of rotation. A maximum of −TI is marked with a downward triangle, and a minimum is marked with an upward triangle. (*c*) TI as a function of the angle of rotation, with the same maximum (downward triangle) and minimum (upward triangle) as in (*b*) indicated with lines in polar coordinates. (*d*) The difference in pixel values between the original image (*a*) and the transformed image corresponding to the local maximum of −TI marked with a downward triangle in (*b*,*c*). (*e*) The difference in pixel values between the original image (*a*) and the transformed image corresponding to the local minimum of −TI marked with an upward triangle in (*b*,*c*). Regions where the original image is higher intensity are indicated in red, while regions where the transformed image is higher are indicated in blue. (Online version in colour.)

Figure 1*a* shows a fivefold regular pattern similar to the ones obtained in [20,22]. We use TI to explore the rotation symmetries of this pattern by applying the algorithm outlined in appendix A. Since the image in figure 1*a* is already stored as an array of intensity values given that we numerically solve equations (2.2) and (2.3), we skip Step (1) in our algorithm since no further processing of the image is required.

For the pattern shown in figure 1*a*, we calculate TI associated with rotations $T_{\theta }$ as a function of the rotation angle θ using equation (2.1) and Step (2) of the algorithm. We take μ=u+v in (2.1) as the sum of the protein concentrations at each location and show a plot of −TI versus rotation angle θ in figure 1*b*. For the parameters studied here, the resulting pattern is centred in the simulation domain, therefore applying Step (1.1) of the method for identifying the coordinates of the image centre is not necessary and does not impact the results of the symmetry analysis.

Figure 1*c* provides an alternate representation of TI in the form of a polar plot (as in Step (4) of the algorithm). In both representations, we mark a peak of −TI (a minimum of +TI) at $\theta =74^{\circ }$ with a downward triangle and a trough of −TI (a maximum of +TI) at $\theta =111^{\circ }$ by an upward triangle (identified as in Step (5) of the algorithm). The pattern remains nearly invariant under the rotation associated with a minimum of TI, as can be seen by the difference in intensities $T_{\theta }\mu -\mu$ shown in figure 1*d*, where blue indicates positive values and red indicates negative values. Such a minimum can be considered an approximate symmetry, with the deviation from perfect symmetry measured by the difference of TI from zero. The local maximum of TI, with $T_{\theta }\mu -\mu$ shown in figure 1*d*, corresponds to rotations by angles which can be considered least symmetric. The difference in intensities are large and the value of TI associated with these approximate antisymmetries can be useful in providing context for interpreting values of the measure associated with symmetries.

## 3. Bilateral symmetry

The proposed method of identifying approximate symmetries can be applied to biological objects which can exhibit a variety of symmetry properties, for instance, objects which possess a left/right or anterior/posterior symmetry. This type of symmetry is called bilateral symmetry, where an object produces two mirror image halves when divided along an axis [42]. For this reason, bilateral symmetry is also called reflection or mirror symmetry. Bilateral symmetry is a very common feature of biological systems, and can be observed in a variety of organisms ranging from bacterial cells and unicellular algae to flowers and humans. In many organisms, bilateral symmetry plays a critical role in important processes such as directed movement [43] and pollination success [42]. A lack of bilateral symmetry can be associated with disease states, and pathological issues during development [44].

**Figure 2.**
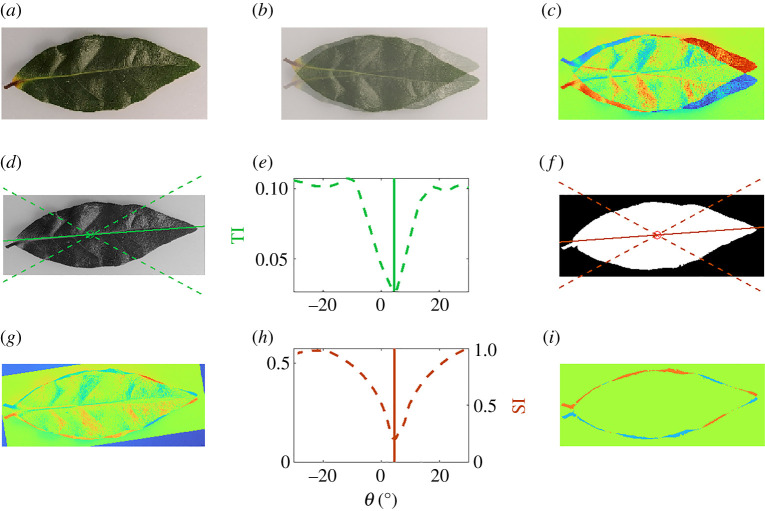
(*a*) An image of a bay leaf (*Laurus nobilis*). (*b*) The original image with its vertical reflection superimposed. (*c*) The difference in greyscale pixel intensity of the reflected and original image. (*d*) The optimal axis of bilateral symmetry as determined by minimizing TI is shown with a solid green line. (*e*) TI bilateral symmetry about axes between the dashed lines in panel (*a*). The optimal axis is marked by a solid vertical line. (*f*) The optimal axis of bilateral symmetry as determined by minimizing SI is shown with a solid orange line. (*g*) The difference in greyscale pixel intensity of the reflected and original image for the optimal symmetry axis indicated in (*d*). Red (blue) indicates higher pixel intensity for the original (transformed) image. (*h*) SI bilateral symmetry about axes between the dashed lines in panel (*f*). The optimal axis is marked by a solid vertical line. (*i*) The difference in area above and below the optimal axis indicated in (*f*). (Online version in colour.)

Measures have been proposed to quantify the deviation between two halves in a bilaterally symmetric object such as a leaf [29,30]. In the Simple Indicator (SI) method, an axis of reflection is manually defined that separates the object into two mirrored halves that are subsequently partitioned by parallel line segments orthogonal to the axis of reflection. The area of the object contained in each partition is calculated independently for the two sides, so that the area in partition i of one side is denoted $A_{i}$ while the area of the other side is denoted $B_{i}$. The SI is then calculated as

$$\text{SI}=\frac{1}{n}\sum_{i=1}^{n}\frac{|A_{i}-B_{i}|}{A_{i}+B_{i}},$$

where n is the number of partitions created by the parallel line segments. This measure captures variation from perfect bilateral symmetry, dependent on the choice of axis and the number of partitions. In the following analysis of a bay leaf (figure 2), we compare the identification of approximate symmetries using SI and TI. We show that with automation of the identification of symmetry axis, both methods produce similar results.

Consider the image of the leaf shown in figure 2*a*, which can be classified as having a strong degree of bilateral symmetry about the central vein. Although this central vein is not straight and the leaf may not be exactly divided along the horizontal in the image, we can attempt to detect the symmetry by considering a reflection about a horizontal line through the centre of the image. The deviation from perfect symmetry becomes more apparent in figure 2*b*, which shows the reflected image superimposed on the original image. Panel (*c*) quantifies the asymmetry using the difference in total pixel intensity between the image and its reflection, with red (blue) indicating higher values for the original (transformed) image.

The difference in pixel intensity shown in figure 2*c* indicates that considering reflections about a slightly tilted axis may provide a better approach for bilateral symmetry. We therefore find a centre point using the algorithm described in appendix A and compute the TI for reflections about an axis with angle θ relative to the horizontal. We apply the algorithm to the greyscale intensity of the original image over a range of angles $\theta \in [-30^{\circ },30^{\circ }]$. Figure 2*d* shows the axis that minimizes TI (solid green) for axes within the dashed lines. The minimum TI is associated to an angle of approximately $4.5^{\circ }$, as shown in figure 2*e*.

In order to compare the TI approach to the area-based SI measure of asymmetry proposed in [29], we also generate the binary image shown in figure 2*f* by thresholding on the greyscale image. We find a central point by equalizing the total area of the leaf above/below and left/right, and consider the SI for axes shown between the dashed orange lines in panel (*f*). The same angle of approximately $4.5^{\circ }$ provides the optimal axis for bilateral symmetry (figure 2*h*), although the centre points found by the two methods are not exactly in the same location.

**Figure 3.**
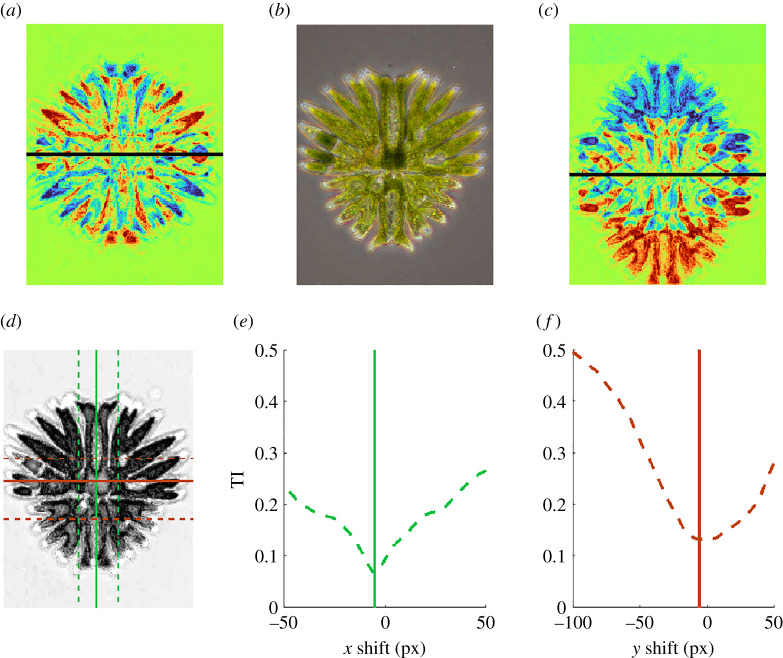
The top row shows an image of the desmid *Micrasteria* undergoing cellular regeneration in the centre panel (*b*). The difference in pixel intensity between its reflection about the solid black line and the original image is shown for an axis through (*a*) the midpoint of the organism, and (*c*) the axis defining its upper and lower structure. The bottom row shows (*d*) the processed image on which TI is computed along with the optimal axes associated with vertical and horizontal reflections in solid orange and solid green, respectively. Reflection TI as a function of the location of the axis relative to the centre of the image is shown for reflections about (*e*) the vertical axis and (*f*) the horizontal axis. In each case, the optimal location that minimizes TI is marked by the solid line. (Online version in colour.)

Another example of bilateral symmetry can be seen in the microscope image of the freshwater unicellular desmid (*Micrasteria*) shown in figure 3*b*. This cell consists of two semi-cells and is regenerating its smaller bottom portion after having previously divided. As with the bay leaf, the bilateral symmetry can be associated with a reflection, this time about a vertical line through the centre of the cell. Although the upper and lower sections of the cell have a similar structure, there is a clear asymmetry between the top and bottom with the top having longer cellular extensions and the bottom still regenerating. As a result, the line dividing the upper structure of the desmid from the lower structure does not fall in the centre of the organism. Thus, the upper and lower halves cannot be easily detected by considering reflection symmetries. The difference between the image of the desmid and its reflection about the central line is shown in panel (*a*). The difference for reflection about the line dividing the upper and lower halves is shown in panel (*c*). Since the differences are larger in panel (*c*), transformation-based methods have difficulty detecting the upper and lower structure in the image.

An axis of bilateral symmetry can be identified by computation of TI as a function of reflections about vertical axes. Given the low greyscale contrast in pixel intensity between the desmid and the background, we use an alternate intensity for μ based on the relative greenness of a pixel:

μ=2g−r−b+1,

where r,g,b are unsigned 8-bit integers that represent the red, green and blue pixel intensity of the original image, respectively. As shown in figure 3*d*, this enhances the intensity of the green desmid and suppresses the intensity of the gray background. The solid green vertical axis minimizes TI for reflections about axes between the dashed green lines, and is therefore identified as the axis of bilateral symmetry. We also use the method to identify the solid orange axis by minimizing TI for horizontal reflections between the orange dashed lines. We note that reflection TI does not identify the axis separating the upper and lower structure of the organism.

The foregoing analysis of bilateral symmetry was restricted to reflections about a straight line. It would be informative to extend the analysis to better capture the shape of the biological centre in cases like the leaf, where the central midvein is curved. Such an extension could be carried out for the area-based measure by computing the area above and below a curve instead of a line. Because TI can be measured relative to any transformation (including nonuniform ones), there are a number of possibilities that would be interesting to explore as a way of extending this measure in a similar way. Spatially dependent transformations may also provide a path towards identification of the upper and lower portions of the desmid, e.g. one could consider transformations consisting of a reflection along with an appropriate rescaling of the portion of the image on one side of the reflection axis. See the discussion of figure 7 in §6 for an illustration of the use of TI with rescaling transformations in the context of fractal patterns in leaf development.

## 4. Rotation symmetry

**Figure 4.**
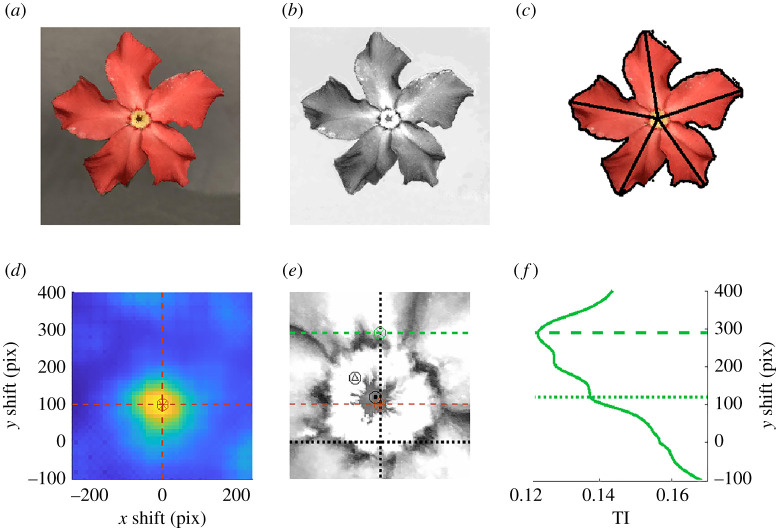
(*a*) An image of a *Pachypodium* flower. (*b*) The processed image on which the TI is being computed. (*c*) Manual identification of the floral edges and distances from the centre to each petal edge. (*d*) A peak in the magnitude ∥dTI/dθ∥ (high values in yellow) for rotations about a fixed location indicates the inferred centre point of the flower shown in (*a*)–(*c*). (*e*) The centre point found in (*b*) based on rotational TI (orange asterix) provides a much better approximation to the manually identified centre (black target) than the centre found by equalizing areas above/below and left/right of the black circled up arrow mark. (*f*) The vertical position of the optimal centre as found by reflection TI (dashed green) is well above the centre points found by other methods. However, a smaller peak in reflection TI, indicated by a dotted line, does appear near the manually identified centre. (Online version in colour.)

The *Pachypodium* flower in figure 4*a* is used here to illustrate the identification of approximate rotation symmetries. The specimen has not been flattened before being photographed, so the non-planar curvature of petals adds to the asymmetry. Figure 4*b* shows the excess of red pixel intensity over the average pixel intensity across the three colour bands. The edge of the flower along with the centre have been manually identified with appropriately tuned thresholding on various combinations of the colour intensities in figure 4*c*. The longest distance from the centre to the petal edge is also marked in panel (c).

The flower has five petals that are approximately evenly spaced and similar in size. There is a clear reflection asymmetry to the petal morphology, and thus one might expect rotational symmetries but not reflection symmetries of an idealized model of the flower.

We explore transformation-based automated methods for identifying the centre of the flower for rotations and reflections as compared to the manual identification shown in figure 4*c*. We compute TI associated to rotations at 12 evenly spaced angles about centre points within a 500 by 500 pixel window near the centre of the flower, and downsample the image by a factor of 16. Figure 4*e* shows the search window within the image, with the middle of the x and y image axes marked by dotted black lines. We find that the norm of the difference in TI for consecutive rotations, shown as a function of centre point in panel (*d*), provides a good measure for the quality of the symmetries for rotations. The motivation to consider this measure is that, for perfect symmetries, TI has very large amplitude of variation as a function of rotation angle (see §2(a) and figure 1). Therefore, the optimal centre will have a large value for ∥dTI/dθ∥. The centre determined in this way, marked by an orange-circled star is close to the manually determined centre indicated by a black target in figure 4*e*. We also compare this to finding the centre by minimizing TI associated to reflections about horizontal and vertical lines passing through the search window, indicated by the green-circled x. Figure 4*e* shows that the horizontal coordinate of the centre is well-approximated, presumably because the flower is aligned nearly optimally for vertical reflections to minimize TI. The vertical coordinate is further off (see green dashed line in figure 4*e*,*f*), since there is no such alignment for vertical reflections. However, we do note a small local minimum in the TI associated to vertical reflections that is near the manually identified centre, indicated with the green dotted line in figure 4*f*. Lastly, we attempted the method of matching areas left/right and up/down, and indicate the result with a circled triangle in the search window. This provides a closer match than the reflection TI, but not as good as the rotation TI measure.

**Figure 5.**
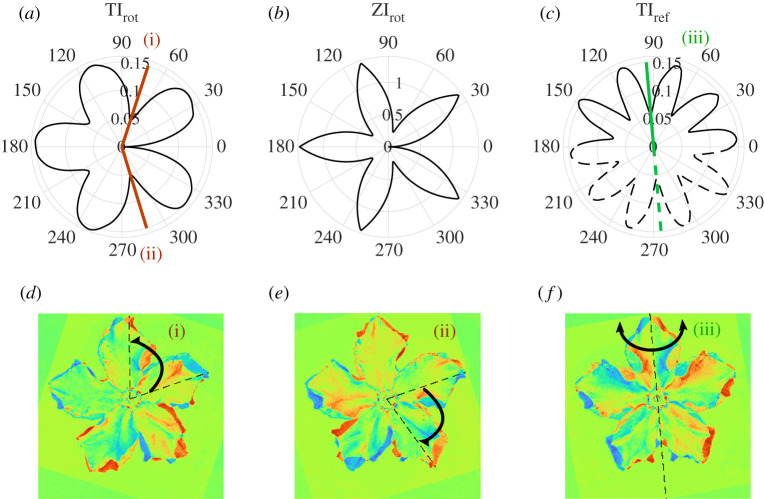
(*a*) Translation information as a function of axis angle for rotations about the manually determined centrepoint of the flower shown in figure 4*a*–*c*. The top ranked symmetries among these are (i) rotation by $72^{\circ }$, (ii) rotation by $-73^{\circ }$. (*b*) The measure in [36] (denoted by ZI), which calculates the sum of the squared distances between nearest petal tips of the original and rotated image. (*c*) Translation information as a function of axis angle for reflections about the manually determined centrepoint of the same flower. The top ranked symmetry among these is (iii) reflection about axis with angle $95^{\circ }$. (*d*) Corresponding difference in pixel intensities between the rotation (i) in (*a*) and the original image. (*e*) Corresponding difference in pixel intensities between the rotation (ii) in (*a*) and the original image. (*f*) Corresponding differences in pixel intensities between the reflection (iii) in (*c*) and the original image. (Online version in colour.)

We compute TI associated to rotations about the identified centre point by an angle θ, as well as reflections about an axis that is an angle θ from the horizontal axis passing through the centre point (figure 5). We compare this to the sum of the squared distances from the petal tips of the original image and the image rotated by an angle θ, an approach inspired by Zabrodsky *et al.* [36] (figure 5*b*). The symmetries detected as minima of this measure (which we denote by ZI) are the rotations associated to exact fivefold symmetry: $72^{\circ }$, $144^{\circ }$, $216^{\circ }$ and $288^{\circ }$. Since each petal has been associated with a single point (farthest on the petal from the centre), some of the asymmetries of the specimen are not captured as well. More details of the flower morphology can be encoded by using more than one landmark point on each petal, but such points must often be manually identified [35]. By contrast, the TI method can be applied directly to the entire image with no manual pre-processing once an appropriate μ has been identified. Moreover, additional information such as the location of the manually identified centre and other landmarks can easily be incorporated into the TI method by manually assigning suitable values of μ at those points.

According to the local minima of the rotations (${\text{TI}}_{\text{rot}}$) and reflections (${\text{TI}}_{\text{ref}}$), the two strongest symmetries of the flower are associated to rotations by the angles of $72^{\circ }$ and $-73^{\circ }$ (figure 5*a*). The difference in pixel intensity between the transformed and original image for these two rotations are shown in figure 5*d*,*e*. Red in these images indicates higher pixel intensity for the original image, and the dashed lines indicate the location of the rightmost petal in the original and transformed images. Although the other rotation angles ($\pm 144^{\circ }$) associated with pentagonal symmetry also have local minima in TI, we find that two reflection symmetries actually have lower TI values than these rotations (figure 5*c*). The difference in pixel intensity between the reflection about $95^{\circ }$ and the original image is shown in figure 5*f*, with the dashed line indicating the axis of reflection.

## 5. Translation symmetry

**Figure 6.**
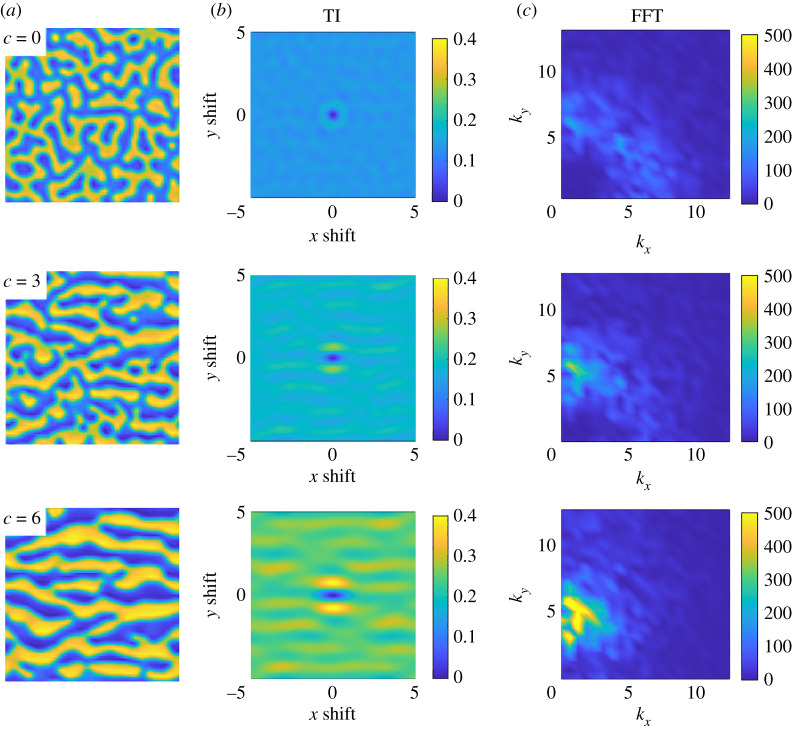
Patterns obtained from the Turing model (2.2), (2.3) with the same random initial condition and an advection term of the form cδ(∂u/∂y) added to equation (2.2). Simulations are carried out on a 10×10-unit periodic domain, with advection rate increasing from top to bottom: c=0,3,6. In each row, the left panel (*a*) shows the spatial distribution of u+v at t=100 with yellow indicating high values. The centre panel (*b*) shows translation TI for the periodically extended state as a function of the magnitude of the vertical and horizontal shifts. The right panel (*c*) shows the Fourier spectrum of the deviation from average of u+v for comparison. (Online version in colour.)

We explore TI in the context of translation symmetry using the Turing model (2.2) and (2.3) on a 10-unit square domain with periodic boundary conditions. We carry out numerical simulations using a fourth-order exponential time differencing scheme in Fourier space [45]. With parameters $\delta =5\times 10^{-3}$, D=0.5, α=1, γ=−1, β=−1, $r_{1}=1$ and $r_{2}=0$, the quadratic terms in the model are not present and stripes are preferred to spots as a result. Starting with a random initial condition, the spatial distribution of u+v is shown at t=100 in the top panel (*a*) of figure 6 (yellow corresponds to high values and blue to low values). The pattern is an irregular combination of labyrinths and patches with no clear preferred spatial direction. Visual inspection indicates a characteristic lengthscale associated with the spacing of the disordered stripes and spots, albeit with significant variation. Linear stability analysis indicates a broad range of unstable modes, with the fastest growing mode having a wavenumber of about 7.25, corresponding to a wavelength of about 1.15. We compute TI as a function of translations in x and y, assuming periodic extensions of the domain. The translation TI, shown in the top of panel (*b*), has a minimum at the centre which corresponds to the identity transformation (TI=0). A peak in TI surrounding this minimum indicates an annulus of least symmetric translations with a radius of about 0.6, and corresponds to shifts by approximately half the characteristic pattern lengthscale in any direction. The top of panel (*c*) shows the absolute value of the Fourier spectrum of the deviation of u+v from its average value. This spectrum, consistent with TI and visual inspection, indicates a characteristic lengthscale with a broad peak at around a wavenumber of magnitude approximately 6, but with no preferred direction.

**Figure 7.**
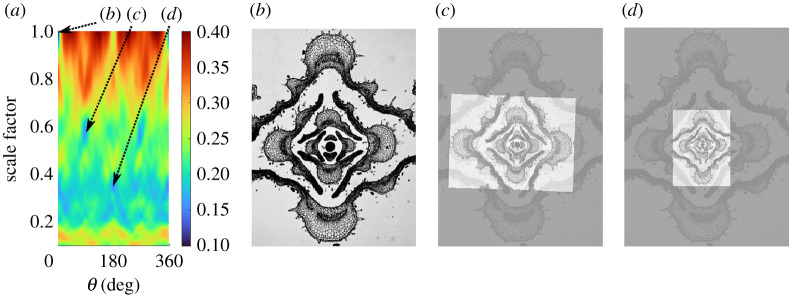
Transformation Information associated with rotation by angle θ and rescaling by a constant factor for a transverse section through a catnip stem bearing pairs of opposite leaves (*Nepeta cataria*). Panel (*a*) shows the location of local minima (with low values in blue) in TI of the image of leaf development shown in panel (*b*). Panels (*c*,*d*) show the original image superimposed, with panel (*c*) rotated by $88^{\circ }$ and rescaled by a factor of 0.57, and panel (*d*) rotated by $180^{\circ }$ and rescaled by a factor of 0.35. (Online version in colour.)

We can induce a preferred direction by introducing a symmetry-breaking advection term of the form cδ(∂u/∂y) into equation (2.2). For larger advection speeds along the y-direction (controlled by c), the pattern generated from the same random initial condition develops into a pattern of stripes that are increasingly aligned along the x-direction. Spatial distributions of u+v with increasing advection parameter c are shown in column panel (*a*) of figure 6. Both the translation TI (column panel (*b*)) and the Fourier spectrum (column panel (*c*)) show an associated increase in signal along the preferred y-direction as c increases.

## 6. Discussion

In this study, we have developed an asymmetry measure based on transformation information (TI) and implemented it as a tool for identifying approximate symmetries in uni- and multicellular biological systems. The TI measure, originally proposed in the context of condensed matter systems, quantifies the difference between an object and its transformation associated with a symmetry of interest. In order to identify a set of approximate symmetries, we search for local *minima* in TI over the set of all transformations associated with potential symmetries. The minima in the TI function correspond to transformations exhibiting the highest degree of symmetry and are, therefore, considered the approximate symmetries with a lower TI value indicating that the symmetry is closer to exact. In addition, TI can be used to identify an optimal axis of symmetry for translation transformations, and optimal coordinates of the centre of the image for rotation transformations. These two features provide an advantage over current symmetry measures. In particular, current measures may introduce user error through identification of symmetry axes and landmark points on the boundary of the object of interest, both of which are determined algorithmically in the approach developed in this work. Importantly, the symmetry properties identified by the new measure were verified and shown to be reasonable for representative biological images and Turing patterns.

We applied the new measure to a range of objects with distinct symmetry properties, including fivefold patterns produced by a Turing model, a bilaterally symmetric algal cell and leaf, and a pentamerous flower with rotational symmetry, thereby demonstrating the flexibility of the measure as well as its ability to reliably identify different types of symmetries. As an illustration that the TI approach applies beyond the symmetries commonly considered by other measures in the literature, we consider the cross-section through a catnip stem shown in figure 7*b*. There is a stem at the centre, with a series of opposing pairs of leaves moving outward in the image. The structure exhibits a fractal symmetry in leaf arrangement associated with rotation and rescaling of the image. Figure 7*a* shows TI for transformations that are a composition of rotation by an angle θ and rescaling by a factor S. The original image, shown in panel (*b*), remains fixed under the identity transformation associated to $\theta =0^{\circ }$ and S=1. Two local minima in TI are superimposed over the original image in panels (*c*), (*d*). Panel (*c*) corresponds to the transformation with $\theta =88^{\circ }$ and S=0.57, and each set of opposing leaves in the transformed image aligns atop the layer of leaves just interior in the original image. The image in panel (*d*) is associated to the transformation by $\theta =180^{\circ }$, S=0.35 and maps each set of leaves two layers inward. The identified symmetries are very close to the transformations with $\theta =90^{\circ }$, S=0.6 and $\theta =180^{\circ }$, S=0.36 that one might expect for an idealized model of the structure.

The potential applications of this measure are broad, and especially suitable for analysis of objects under different conditions, e.g. TI can be used to quantify (i) the extent to which organic structures (such as leaves and flowers) manifest phenotypic plasticity in response to different environmental conditions, (ii) morphometric changes during ontogeny and growth, (iii) phenotypic transformations attending evolutionary transitions, (iv) pheno-type genetic variants within populations and (v) assessing pathological conditions.

Symmetry is a ubiquitous feature of organic systems, and is often correlated with fecundity, survival and evolvability. The measure proposed here provides a robust and rigorous method for identifying approximate symmetries in any object, providing insights into fundamental structures and their properties. In addition, it can provide insights into the establishment of polarity through symmetry breaking in biological structures. It is worth noting that this measure provides an accurate description of symmetry phenomena in biological objects, but does not currently identify the corresponding symmetry-breaking mechanisms. While we primarily use the Turing reaction–diffusion process as an example of a pattern formation mechanism here, many other mechanisms have been identified and studied in the context of biological development, i.e. mechanochemical [46], local adhesion gradients [47], minimization of repulsive energy [48]. For instance, both starfish and rosid flowers develop a pentamerous radial symmetry, however very different mechanisms generate symmetry in these systems (holoblastic radial cleavage and spiral phyllotaxy, respectively). Future work will explore the time-evolution of approximate symmetries in biological development, in combination with modelling efforts, as an approach for probing underlying symmetry-breaking mechanisms [49].

## Data accessibility

Sample code for analysing an example image are provided in the Github repository [50].

## Appendix A. Transformation information algorithm for identifying symmetries in biological images

The following steps describe a simple application of the transformation information measure to biological images with symmetries of interest. The method is implemented in MATLAB R2020b (https://www.mathworks.com/) and sample code is provided in [50].

(1) Read the image in Matlab using imread and either convert it into greyscale or extract the appropriate rgb channel from the resulting numeric array.
(2) Measure rotational symmetries by sampling over the rotation angle θ (in some of our examples, θ=k(π/180) with k=0,6,…,359) and by generating the transformed image for rotation by angle θ.
  (a) Define the matrix for the rotation by angle θ (measured counterclockwise from the x-axis): $A_{\text{rot}}(\theta )=\left(\begin{array}{ccc} \cos(\theta ) & -\sin(\theta ) & 0\\ \sin(\theta ) & \cos(\theta ) & 0\\ 0 & 0 & 1 \end{array}\right)$.
  (b) Convert the rotation matrix into a two-dimensional affine geometric transformation object using affine2D.
  (c) Apply the geometric transformation to the object using affineOutputView and imwarp.
  (d) Calculate the transformation information measure TI using (2.1). The integral is evaluated by summing the integrands over all pixel values that are in the domain of both the original and the transformed image.
(3) Measure reflection symmetries by sampling over the reflection axis angle θ as above and by reflecting the image about the axis going through the centre of the image at angle θ.
  (a) Define the matrix for the reflection about the angle θ (measured counterclockwise from the x-axis): $A_{\text{ref}}(\theta )=A_{\text{rot}}(\theta )A_{\text{ref}}A_{\text{rot}}{\left(\theta \right)}^{-1}$, where $A_{\text{ref}}$ is the matrix for the standard reflection in x (i.e. about the y-axis): $A_{\text{ref}}(y)=\left(\begin{array}{ccc} -1 & 0 & 0\\ 0 & 1 & 0\\ 0 & 0 & 1 \end{array}\right)$.
  (b) Follow steps (b)–(d) in Step (2) above.
(4) Visualize the transformation information measure (TI) as a function of the angle θ for the rotation and reflection symmetries in Steps (2) and (3) using polar.
(5) Decide on a number N of symmetries of interest and identify these symmetries by finding the minima of TI for each transformation in Steps (2) and (3). This can be done efficiently by finding and ranking the N most prominent peaks of −TI using findpeaks. MATLAB provides additional documentation regarding how the findpeaks function measures the prominence of peaks in signal data [51].
(6) Extract the angles associated with the top N ranked symmetries and visualize them by applying the appropriate rotations and reflections of the image.

As detailed in the examples in the main text, it is sometimes useful to determine the most appropriate choice for the centre coordinates of certain biological images after step (1) in the above algorithm. One option is to use a similar transformation information approach to determine the translations along the x- and y-axes that best centre the image for further analysis of its symmetries. Below we describe finding the centre of an image using translations and reflections, however a similar approach can be applied to find the centre coordinates using translations and rotations, which could give better results depending on the application.

(1.1) Determine the centre coordinates of the image using the transformation information measure.
  (a) Decide on a range of pixels to test shifting the image along the x- and y-axes by.
  (b) Define the matrix for translating the image along the x-axis by each number of pixels dx in the relevant range: $A_{t}(\text{d}x)=\left(\begin{array}{ccc} 1 & 0 & 0\\ 0 & 1 & 0\\ \text{d}x & 0 & 1 \end{array}\right)$. Apply the reflection about the y-axis and the translation by dx pixels using $A_{\text{shift}}(\text{d}x)=A_{t}{\left(\text{d}x\right)}^{-1}A_{\text{ref}}(y)A_{t}(\text{d}x)$. Note that the order in the matrix multiplication is consistent with the affine transformation framework used by Matlab. Calculate the transformation information measure for each shift as in Step (3) above.
  (c) Similarly, define the corresponding matrix for translating the image along the y-axis by each number of pixels dy in the relevant range: $A_{t}(\text{d}y)=\left(\begin{array}{ccc} 1 & 0 & 0\\ 0 & 1 & 0\\ 0 & -\text{d}y & 1 \end{array}\right)$. Apply the reflection about the x-axis and the translation by dy pixels using $A_{\text{shift}}(\text{d}y)=A_{t}{\left(\text{d}y\right)}^{-1}A_{\text{ref}}(x)A_{t}(\text{d}y)$. Calculate the transformation information measure for each shift as in Step (3) above.
  (d) Find peaks in −TI (as a function of axis shift) and identify the most prominent one as in step (5).
  (e) Determine and record the coordinates of the centre from the most prominent peak in −TI.
